# Impact of Oral Typhoid Vaccination on the Human Gut Microbiota and Correlations with *S*. Typhi-Specific Immunological Responses

**DOI:** 10.1371/journal.pone.0062026

**Published:** 2013-04-24

**Authors:** Emiley A. Eloe-Fadrosh, Monica A. McArthur, Anna M. Seekatz, Elliott F. Drabek, David A. Rasko, Marcelo B. Sztein, Claire M. Fraser

**Affiliations:** 1 Institute for Genome Sciences, University of Maryland School of Medicine, Baltimore, Maryland, United States of America; 2 Center for Vaccine Development, University of Maryland School of Medicine, Baltimore, Maryland, United States of America; 3 Department of Microbiology and Immunology, University of Maryland School of Medicine, Baltimore, Maryland, United States of America; 4 Department of Medicine, University of Maryland School of Medicine, Baltimore, Maryland, United States of America; Argonne National Laboratory, United States of America

## Abstract

The resident microbial consortia of the human gastrointestinal tract play an integral role in modulating immune responses both locally and systemically. However, detailed information regarding the effector immune responses after vaccine administration in relation to the gastrointestinal microbiota is absent. In this study, the licensed oral live-attenuated typhoid vaccine Ty21a was administered in a clinical study to investigate whether oral immunization resulted in alterations of the microbiota and to identify whether a given microbiota composition, or subsets of the community, are associated with defined *S*. Typhi-specific immunological responses. The fecal microbiota composition and temporal dynamics were characterized using bacterial 16S rRNA pyrosequencing from individuals who were either immunized with the Ty21a typhoid vaccine (n = 13) or served as unvaccinated controls (n = 4). The analysis revealed considerable inter- and intra-individual variability, yet no discernible perturbations of the bacterial assemblage related to vaccine administration were observed. *S*. Typhi-specific cell mediated immune (CMI) responses were evaluated by measurement of intracellular cytokine production using multiparametric flow cytometry, and humoral responses were evaluated by measurement of serum anti-LPS IgA and IgG titers. Volunteers were categorized according to the kinetics and magnitude of their responses. While differences in microbial composition, diversity, or temporal stability were not observed among individuals able to mount a positive humoral response, individuals displaying multiphasic CMI responses harbored more diverse, complex communities. In line with this preliminary observation, over two hundred operational taxonomic units (OTUs) were found to differentiate multiphasic and late CMI responders, the vast majority of which classified within the order Clostridiales. These results provide an unprecedented view into the dramatic temporal heterogeneity of both the gut microbiota and host immune responses.

## Introduction

*Salmonella enterica* serovar Typhi (*S*. Typhi), the causative agent of typhoid fever, is responsible for over 20 million illnesses and >200,000 deaths worldwide, and continues to be a major health concern in developing countries, particularly because of the emergence of antibiotic-resistant strains [1], [2]. Currently, two licensed vaccines are available for the prevention of typhoid fever, the live attenuated oral vaccine Ty21a and the parenteral Vi polysaccharide vaccine based on the *S*. Typhi Vi antigen [1], [3]. In endemic areas, particularly south-central and south-east Asia, typhoid vaccines are not widely used mainly due to cost constraints [4]. As described previously, immunization with Ty21a elicits mucosal and serum antibodies, and B memory cells against *S*. Typhi antigens (e.g., lipopolysaccharide O, H antigen), as well as a wide array of specific cell-mediated immunological (CMI) responses [5], [6], [7], [8], [9], [10]. CMI responses are believed to play a key role in the host's defense against this intracellular bacterium by several mechanisms (e.g., cytokine production, killing of infected cells) and detailed kinetic studies have shown that multiphasic CMI responses are characteristic in responders following Ty21a vaccination [4], [7], [11]. However, the precise immune correlates that mediate protection from disease remain largely undefined. The overall protective efficacy of Ty21a has been demonstrated to range substantially from 35–96% in field studies depending on the formulation, dosing schedule (three to four doses are necessary for vaccination with Ty21a), and duration of follow-up post-vaccination [4], [11].

Significant discrepancies exist in the efficacy of oral vaccines in geographically distinct human populations, particularly those in developing countries most susceptible to infection. While socioeconomic situations, host genetics, nutritional state, and exposure to related infectious agents have been suggested as the main contributing factors in vaccine efficacy disparity [12], one of the least explored elements is the composition of the intestinal microbiota. It has been well established that certain members of the resident gastrointestinal microbiota are intricately linked to the production of mucosal immunity [13]. A prime example is that of the segmented filamentous bacteria (SFB), within the order Clostridiales, that promote T helper 17 (T_H_17) cell differentiation in a murine model system [13], [14]. Additionally, in a recent study, the administration of a prebiotic fructooligosaccharide/inulin mix was shown to enhance efficacy of *S*. Typhimurium SL1479 vaccination in a murine model, suggesting that modulation of the resident gastrointestinal microbiota may confer increased mucosal immunity [15]. Similarly, a randomized clinical trial evaluating the humoral immune responses after Ty21a vaccine administration in individuals receiving the well-characterized probiotic *Lactobacillus* GG or placebo demonstrated an increase in specific IgA for those receiving the probiotic [16]. Together, there appears to be at least circumstantial evidence that the resident gastrointestinal microbiota can be modulated to enhance vaccine efficacy. However, to date, no comprehensive studies have been undertaken to examine the gastrointestinal microbiota in relation to vaccine administration and if there is a discernible alteration in the community following vaccine administration. The fundamental question is whether a particular microbial community composition is associated with greater vaccine efficacy. A critical breakthrough in our understanding of immunization, leading to more efficient development of vaccines, could be made if a ‘responder’ gastrointestinal microbiota can be identified, particularly for those immune responses that correlate with protection.

In this study, we present a cultivation-independent longitudinal survey of the fecal bacterial assemblage from individuals who were orally immunized with the licensed live-attenuated Ty21a typhoid vaccine (n = 13) or served as unvaccinated controls (n = 4). Phylogenetic profiling of the bacterial community was utilized to determine whether there were measureable alterations in the gut microbiota in response to Ty21a vaccination, as well as to evaluate whether a given composition, or subsets of the community correlated with induction of effector immune responses.

## Results and Discussion

### Characteristics of the fecal bacterial 16S rRNA phylogenetic profiles

**Figure 1:**
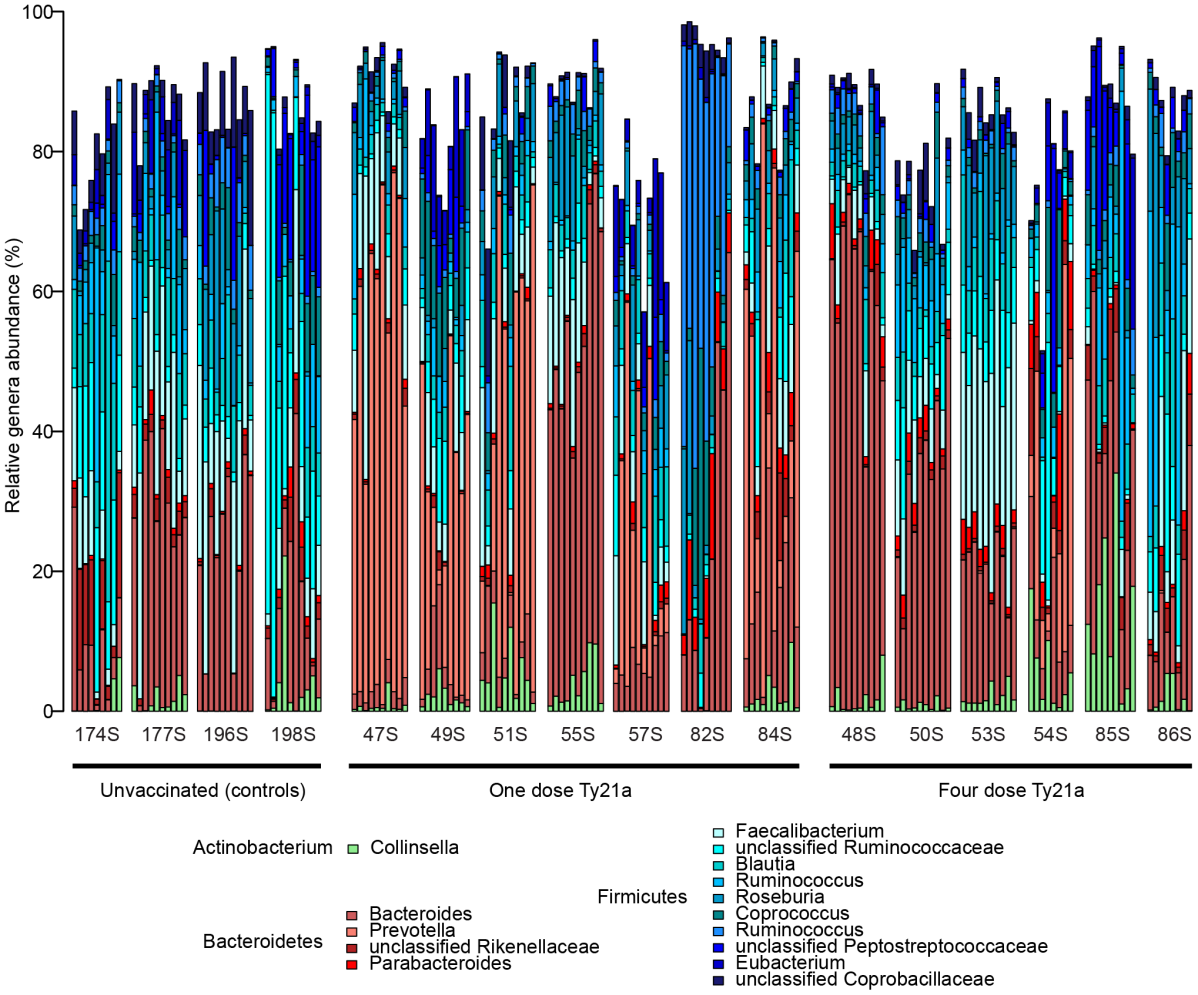
Phylogenetic profile of dominant bacterial genera for all volunteers. Stacked bar charts in chronological order for each volunteer of the 15 main genus-level bacterial groups identified based on ≥2% abundance present in at least two samples. Unclassified sequences are not shown.

The fecal microbiota was characterized by 454 pyrosequencing of bacterial 16S rRNA gene amplicons (V1–V2 region) from stool samples collected longitudinally from seventeen individuals (clinical study design and volunteer description, Table S1). Stool samples were collected a week prior to immunization (day -7), the day of immunization (day 0), and at 8 time-points after immunization (days 2, 4, 7, 10, 14, 28, 42, 56). A total of 689,149 high-quality sequences were generated, corresponding to an average 4,254 reads per sample with an average length of 254 base pairs. Using the naïve Bayesian classifier with the Greengenes database implemented within mothur [17], [18], a total of 164 genera were identified from 13 separate phyla, with 15 of these genera appearing at ≥2% abundance in at least two samples (Fig. 1). Consistent with previous studies of the human fecal microbiota [19], the phyla Firmicutes and Bacteroidetes represented the vast majority of classified sequences with abundances of 58% and 34% across all samples, respectively. Within the phylum Firmicutes, members of the class Clostridia constituted ∼40% of the classified sequences distributed among the genera *Faecalibacterium* (10%), *Roseburia* (7%), *Coprococcus* (5%), *Blautia* (5%), and three different clades within *Ruminococcus* (12%). Although the Firmicutes were the dominant phyla, the genera *Bacteroides* and *Prevotella*, members of the phylum Bacteroidetes, represented the greatest relative abundances of classified sequences across all samples at 21% and 12%, respectively (Fig. 1, Fig. S1).

**Figure 2:**
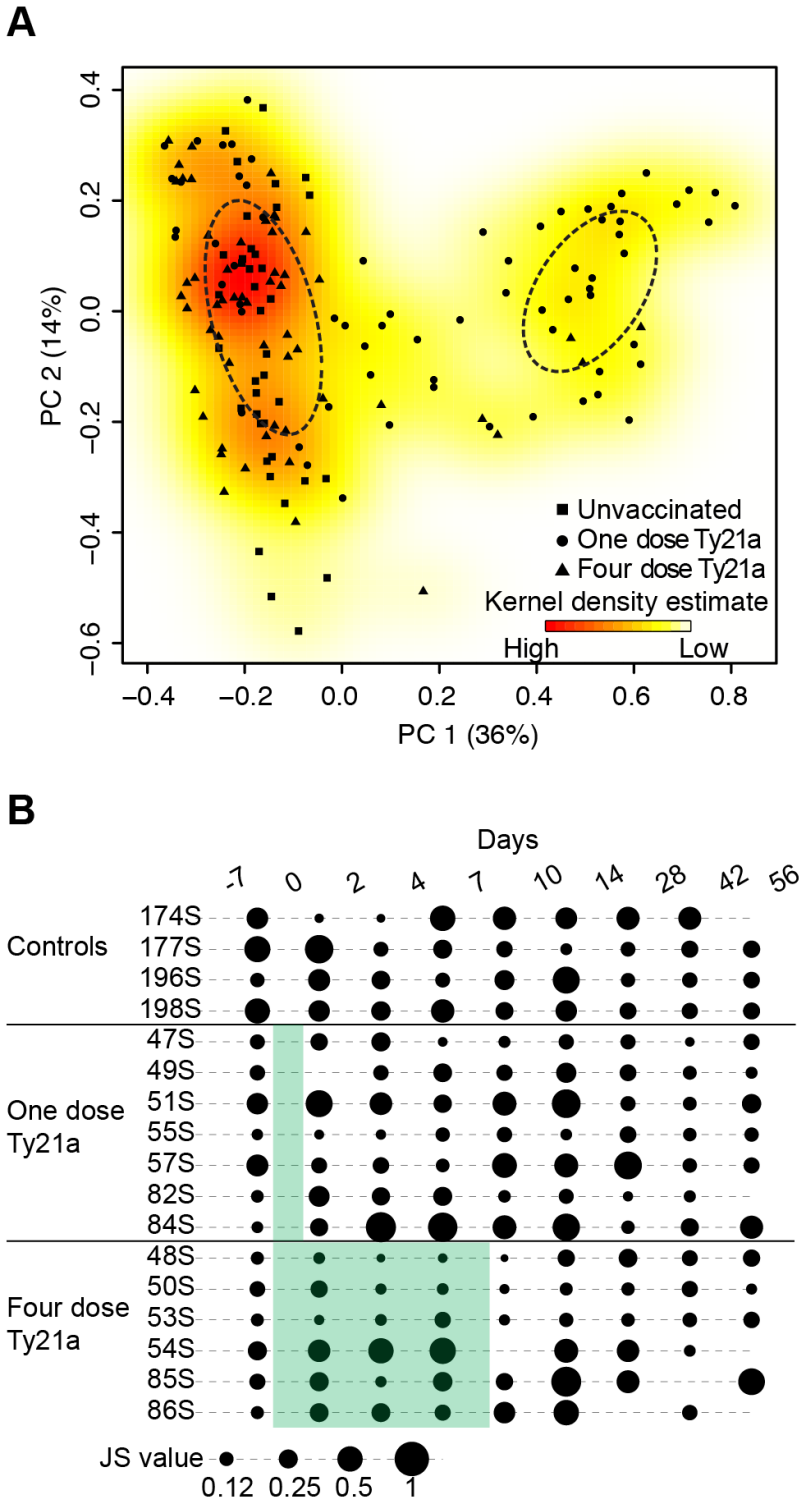
Community composition and structure for all samples over time. (**A**) Principle coordinate analysis (PCoA) based on the Jensen-Shannon divergence for all samples, colored by the multivariate kernel density estimation with vaccination groups designated by unvaccinated control (▪), one dose Ty21a (•), and four dose Ty21a (Δ). Dashed-line ellipses are fitted around the two ‘community type’ clusters, the area of which is calculated from the standard deviation of all points classified within one of the two clusters. (**B**) Longitudinal dot plots of Jensen-Shannon (JS) divergence values between consecutive time points for the seventeen individuals. Dot area corresponds to JS divergence values and is represented in the scale at the bottom of the figure. Shaded green area represent Ty21a vaccination schedule for volunteers receiving one dose (t = 0) and four doses (t = 0, 2, 4, and 7 days).

To identify samples harboring similar microbiota community composition, we implemented multidimensional cluster analysis based on the Jensen-Shannon divergence (Fig. 2). Two ‘community type’ clusters, or ‘enterotypes,’ were identified and validated based on the criteria of Arumugam et al. [19]. However, the average silhouette width for two clusters (*S(i)* = 0.44; 3 clusters *S(i)* = 0.28) was only weakly supportive of a defined clustering structure [20], with a continuous gradient reflected in the multivariate kernel density estimation (Fig. 2A). As opposed to a stratified, discrete clustering of the gastrointestinal microbiota as first proposed by Arumugam et al. [19], we observed a more continuous gradient within the present cohort defined chiefly by the relative abundances of the genera *Prevotella* and *Bacteroides* within the phylum Bacteroidetes. These two genera appear to be mutually exclusive within an individual's fecal microbiota at any given time point, with the majority of samples identified as a *Bacteroides*-dominated ‘community type’ (n = 123) compared to the *Prevotella*-dominated ‘community type’ (n = 39) (Fig. S1). Wu et al. [21] suggest that the ‘community types’ may be partitioned by dietary constraints, where the *Bacteroides*-dominated ‘community type’ is associated with animal protein and saturated fats from a Westernized diet, while the *Prevotella*-dominated ‘community type’ is associated with a carbohydrate-based diet. No dietary information, however, was collected for the present cohort to provide additional support for the patterns of *Prevotella* and *Bacteroides* abundances observed.

Strikingly, consecutive longitudinal analysis for the seventeen individuals across ten discrete time points demonstrated considerable variability with respect to community similarity (Fig. 2B). No consistent pattern was observed for differences in Jensen-Shannon divergence values among individuals for a given time interval, nor were these differences correlated to vaccine administration schedule. For example, volunteer 48S (receiving four doses Ty21a) maintained a highly consistent fecal community over time, while volunteer 174S (unvaccinated) displayed considerable variability from one time point to the next (Fig. 2B, Fig. S2). These findings are congruent with previous studies. Marked overall variability in an individual's microbiota across a wide temporal sampling from months, weeks and days has been observed for two individuals sampled from four body sites [22]. Jalanka-Tuovinen et al. [23] also identified temporal stability of the ‘core’ members of the microbiota with much of the variation attributed to ‘non-core,’ transient phylotypes. We extend and qualify these observations to reflect that the dynamics and variability of a given microbiota sampled over time is highly individual, where wide variability or maintenance of similar composition between sampling times is displayed.

### Fecal community composition and structure over time reveals intra-individual variability irrespective of vaccine administration

General ecological theory suggests diversity is positively related to ecosystem stability, whereby stability depends on functional redundancy or the ability of a subset of species, or functional groups, to differentially respond to perturbations [24]. In this context, vaccination with the Ty21a oral typhoid vaccine was hypothesized to act as a perturbation that would be reflected in the fecal community composition and structure as a decrease in diversity. Community richness, phylogenetic and OTU diversity were tested to evaluate whether vaccination with Ty21a disrupts (destabilizes) the microbiota. The Shannon diversity index (*H*), a measure accounting for both species abundance and evenness, was compared across time for the unvaccinated (controls), one-dose, and four-dose volunteer groups using the normalized OTU profiles. The Simpson inverse diversity index (1/*D*) was additionally utilized for comparisons, taking into account community richness, abundance, and is less sensitive to rare OTUs compared to the Shannon diversity index. Lastly, phylogenetic diversity (PD), a separate measure of community diversity based on the taxonomic relatedness using a phylogenetic tree was used to compare among sample groups and time points.

**Figure 3:**
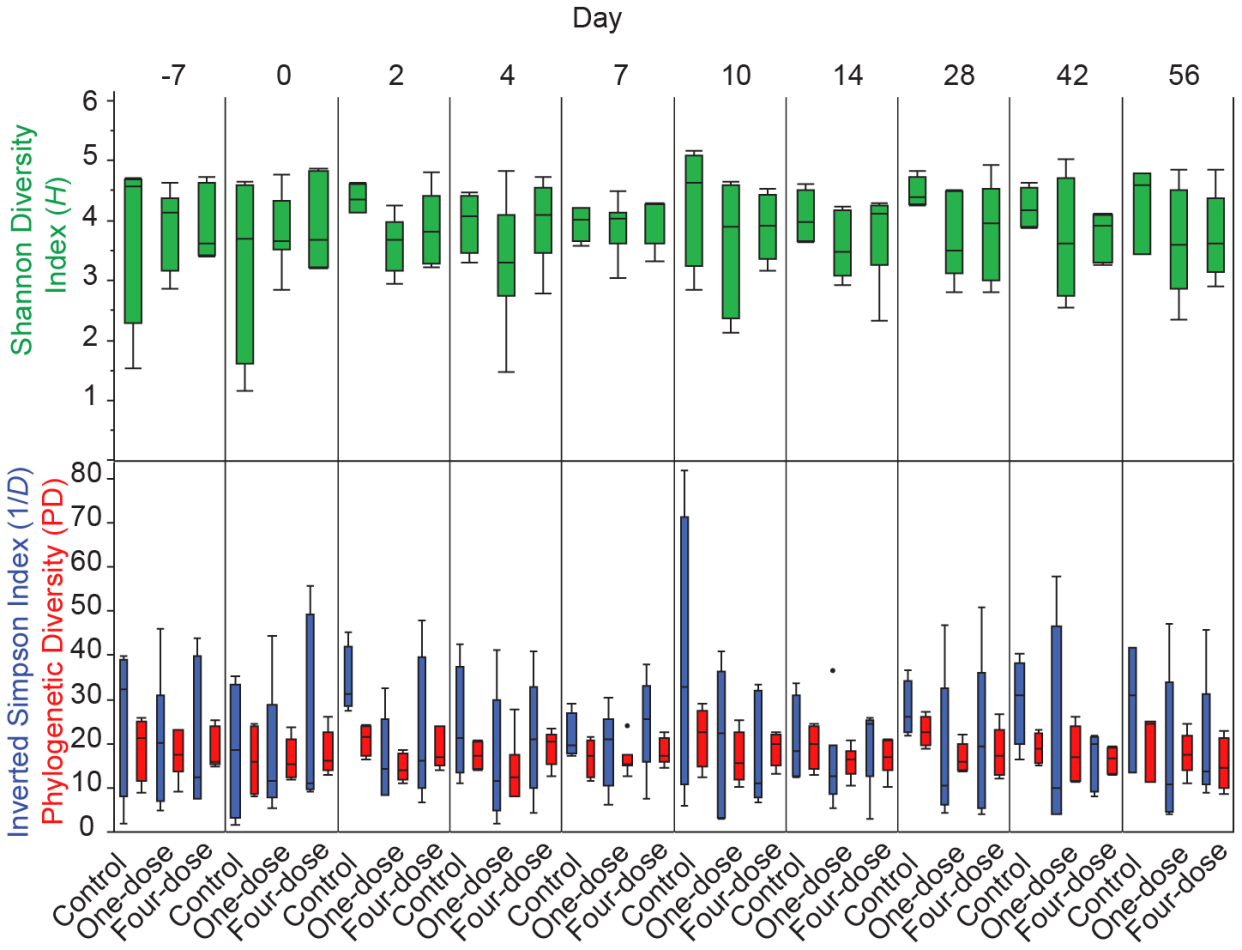
Community richness and diversity over the course of vaccine administration. Samples were divided by time for the unvaccinated, one-dose, and four-dose volunteers and tested for differences in OTU diversity and richness (Shannon diversity (green) and Inverted Simpson diversity (blue) indices), as well as Phylogenetic diversity (red, PD). All samples were rarefied to the same number of reads per sample (1,011 reads/sample) to account for uneven sampling.

Overall, the unvaccinated and vaccine treatment groups were remarkably stable over the sampling period as reflected by all three diversity indices (Fig. 3). The nonparametric Wilcoxon rank sums test for multiple pairwise comparisons was employed to evaluate whether a difference in means over time was evident. While fluctuations in individual diversity measurements were apparent, this analysis revealed no statistically significant differences in any of the means for Shannon diversity, inverted Simpson diversity, and PD over time and among the three volunteer group microbiota communities. Additionally, there was no significant difference in diversity corresponding to the days during vaccine administration (one-dose volunteers, day 0; four-dose volunteers, days 0, 2, 4, 7). Therefore, our initial hypothesis that vaccination would perturb the fecal bacterial assemblage as measured using 16S rRNA community profiling and assessed by three separate diversity measures was not supported. Analysis of a larger cohort of individuals is needed to confirm the validity of these results; however, the current study confirms that the decreased reactogenic properties of the vaccine selected for during the development of the Ty21a vaccine appears to include the lack of perturbation of the microbiota. Additional studies to examine the functional diversity and redundancy of the microbiota (e.g., whole-community metagenomic or transcriptional profiling of the active microbiota) are required.

### Humoral and cell mediated immunological responses and kinetics similarly display inter-individual variation

In an effort to delineate the interplay between the human host, resident microbiota and oral immunization with the Ty21a vaccine, humoral and CMI responses (Tables S2 and S3, respectively) were measured at the same times as described for the microbiota community sampling. Using multi-parametric flow cytometry methods capable of detecting simultaneously five intracellular cytokines/chemokines (interleukin (IL)-17A, IL-2, interferon (IFN)-γ, tumor necrosis factor (TNF)-α, and macrophage inflammatory protein (MIP)-1β) [10], CD8^+^ T cell measurements were collected for two unvaccinated volunteers (177S and 196S) and the six volunteers receiving four doses of Ty21a (48S, 50S, 53S, 54S, 85S and 86S). Given the complexity of the measurements and expense of performing these detailed CMI determinations, we focused the studies of associations between CMI and microbiota measurements in volunteers receiving the full vaccination schedule which has been shown to elicit protection in the majority of vaccinees [4], [11]. CMI responses against *S.* Typhi have been studied extensively and multiphasic (bi- or tri-phasic) responses are characteristic [8], [10]. The kinetics of multifunctional and multiphasic responses for four of the 4-dose volunteers have been previously described [10]. Based on the kinetics and magnitude of intracellular net IFN-γ production (time-point after immunization minus day 0), volunteers were grouped as multiphasic responders (50S, 53S, and 86S), late responders only (54S and 85S), or non-responders (48S) (Table S3). IFN-γ production was chosen as an indicator of responsiveness because this is the cytokine that has been most extensively studied in CMI responses against *S*. Typhi [5], [6], [7], [8], [9], [10]; however, when the analyses were performed based on TNF-α or IL-2, the same grouping was observed. Only one of the six four-dose volunteers (48S) for whom microbiota data was available was a non-responder (no cytokine production greater than 1% over baseline values) (Table S3). Multiphasic response was defined as IFN-γ production greater than 1% over baseline before day fourteen post-immunization followed by an additional peak or peaks in IFN-γ production fourteen days or later post-immunization. Late response was defined as at least one-time point with IFN-γ production greater than 1% over baseline greater than day fourteen post-immunization only. Two of the three multiphasic responders were extremely high responders (50S and 53S) and exhibited IFN-γ production greater than 5 times the level of the negative controls (Table S3). Additionally, serum antibody responses (IgA and IgG against *S*. Typhi LPS) were measured for all individuals (Table S2). Antibody responses were considered positive if there was a 4-fold or greater increase above values at day 0 at one or more time-points after immunization. As with the microbiota, there was significant heterogeneity observed in the CMI and humoral immune kinetics and responses to Ty21a immunization.

### Categorical analysis reveals bacterial taxa differentially represented in individuals displaying multiphasic CMI responses

**Figure 4:**
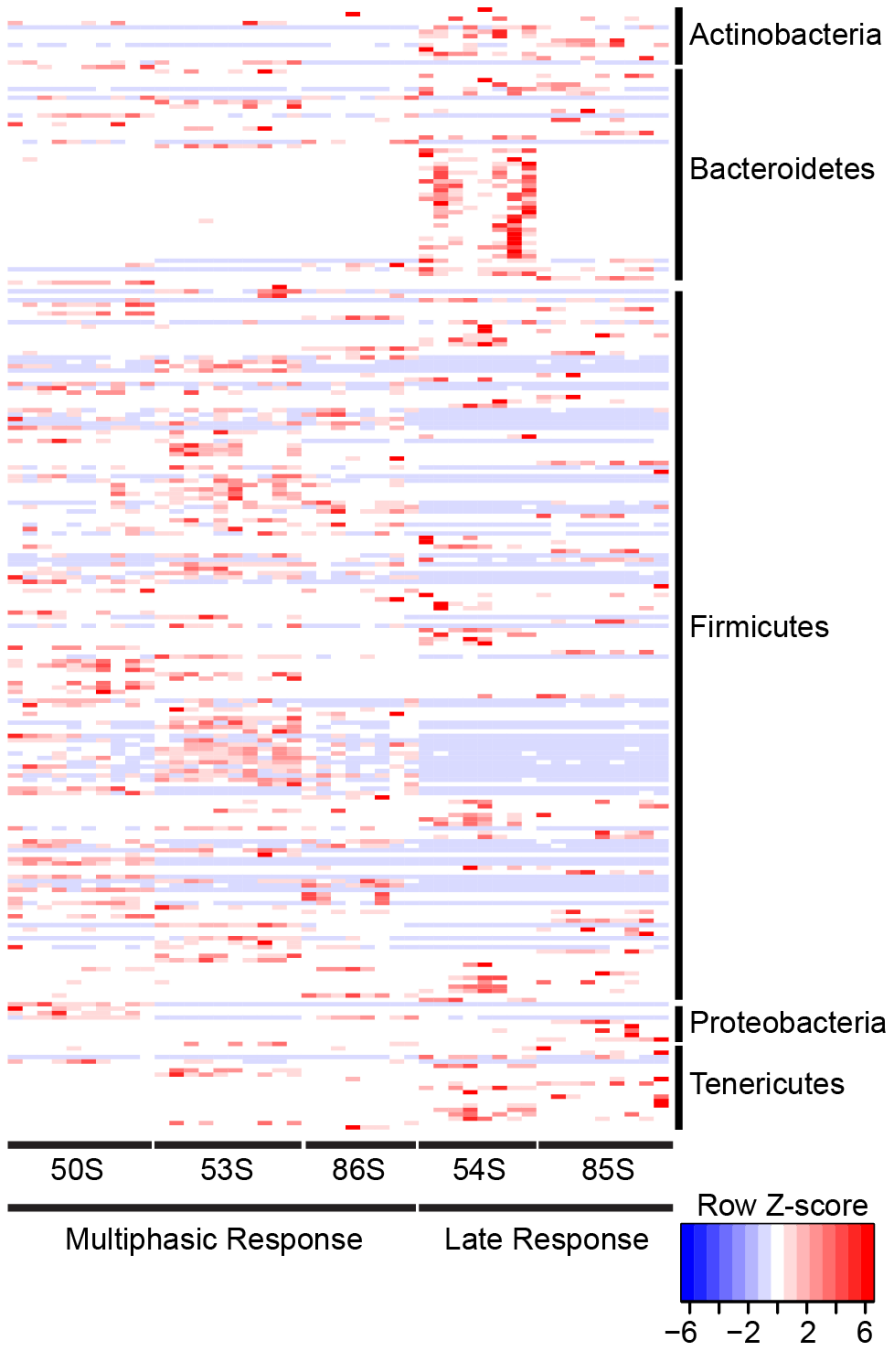
Differentially abundant OTUs identified between multiphasic CMI responders and late only CMI responders. OTUs are ordered by consensus taxonomic classification, with OTUs scaled by relative abundances for each row ranging from low relative abundance (blue) to high relative abundance (red).

We next implemented a categorical analysis [25] to evaluate whether specific bacterial OTUs discriminated between individuals displaying multiphasic CMI responses (50S, 53S, 86S) compared to late CMI responses (54S and 85S). Over two hundred bacterial OTUs were identified as differentiating the three multiphasic CMI responders from the two late CMI responders (Fig. 4, Table S4). Differential Bacteroidetes OTUs were associated almost exclusively with subject 54S. The significance of this finding is at present unknown. Intriguingly, the majority of differential OTUs (161 OTUs, 63% total) classified within the order Clostridiales, predominantly within the families Lachnospiraceae and Ruminococcaceae. Atarashi and colleagues [26] recently demonstrated that resident Clostridium species in mice are effective inducers of colonic regulatory T-cells. Similarly, it is well established that the mouse Clostridiales-classified segmented filamentous bacteria (SFB) are potent activators of intestinal immune responses [13], [14], [27]. It should be noted, however, that the polyphyletic grouping using 16S rRNA-based taxonomy confounds taxonomic resolution within the order Clostridiales, and is further compounded by inconsistencies in assigning phylogenetic affiliation based on morphological and/or biochemical properties [28], [29]. Future work to delineate human-associated immune-modulatory members of the Clostridiales will be critical.

**Figure 5:**
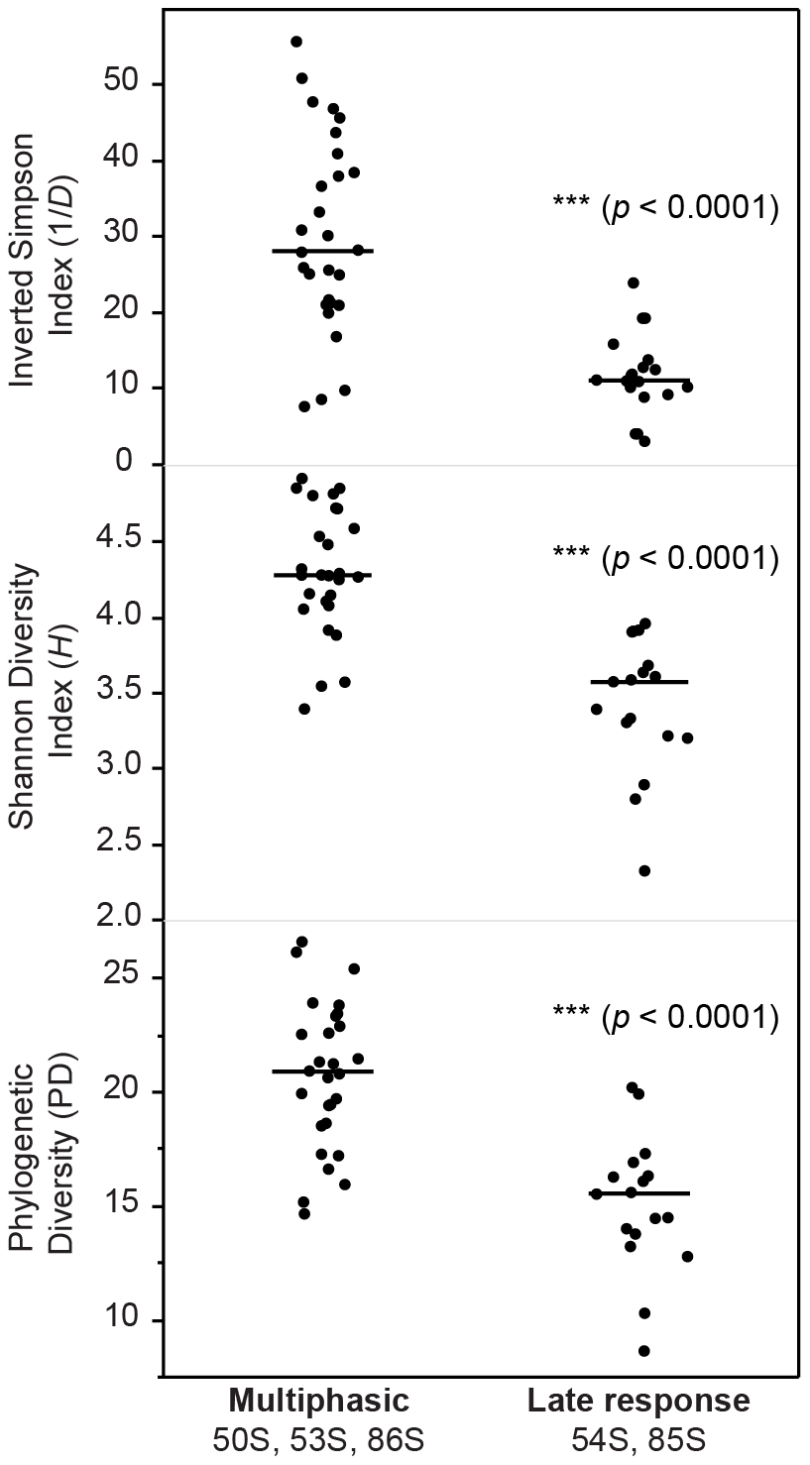
Differences in community diversity among multiphasic CMI responders and late CMI responders. Shannon diversity, inverted Simpson diversity, and phylogenetic diversity (PD) were calculated from the rarefied OTU data. The bars represent the mean diversity values. By all measures, the multiphasic CMI responders were significantly greater compared to the late only CMI responders.

The results suggest that a subset of bacterial taxa might generally discriminate among individuals capable of mounting a multiphasic immunological response to Ty21a compared to individuals mounting a late response. Clearly, a more thorough evaluation of individuals encompassing a more extensive cohort is essential to give statistical power to this initial finding. Moreover, community diversity was compared for multiphasic CMI responders (50S, 53S, 86S) and late only CMI responders (54S and 85S) to test whether overall community diversity distinguished between the two groups. With the caveat of small sample size, we identified that multiphasic CMI responders harbored significantly greater diversity in all three measures of community diversity compared to the late CMI responders (Fig. 5). A distinct relationship between decreased gut microbial complexity in individuals with gastrointestinal disorders, such as inflammatory bowel diseases (IBD) compared to healthy individuals, has been previously demonstrated [30]. It is reasonable to hypothesize that a complex and diverse gastrointestinal bacterial assemblage could be associated with an individual's temporal heterogeneity in cell mediated immunological response to vaccination.

Despite the findings of distinct community diversity between volunteers based on CMI responses, no significant differences in overall community diversity were identified between humoral responders and non-responders (Fig. S3). It is important to note that these results are based on serum IgG and IgA antibody titers, which may not reflect the humoral response at the mucosal level. The role of serum anti-*S*. Typhi antibodies in protection remains unclear. However, there is substantial evidence that while humoral responses may play a part in protection against *S*. Typhi, they probably do not represent the dominant protective immune response (reviewed in [7]).

These analyses are limited by the number of individuals recruited. Future studies involving additional vaccinated and unvaccinated subjects will be required to confirm and extend the statistically significant findings described in this manuscript. Nevertheless, these initial results provide invaluable information regarding the impact of Ty21a vaccination on the gut microbiota and potential discriminatory bacterial taxa, which correlated with heterogeneous CMI responses elicited after vaccination. Furthermore, the longitudinal study design affords fundamental insights into both the gut microbiota and immunological responses.

### Summary

There is growing support from clinical trials in developing countries that the gastrointestinal microbiota can impact the efficacy of a given vaccine [12], as well as evidence in animal models that modulation of the gastrointestinal microbiota (e.g., with antibiotics, prebiotics, and probiotics) can enhance vaccine efficacy [15], [16]. The present study represents a detailed examination of the interplay between the human host and members of the fecal bacterial community, and the impact of live oral vaccination across a comprehensive longitudinal sampling. Immunization with either a single dose or four-dose vaccination schedule of Ty21a did not appear to disrupt the composition, diversity or stability of the bacterial community. Categorical analysis based on multiphasic CMI responses (50S, 53S, 86S) and late CMI responses only (54S and 85S) identified a subset of bacterial OTUs differentiating those individuals capable of mounting a heterogeneous immunological response. Generally, individuals exhibiting a multiphasic immune response to vaccination harbored greater community richness and diversity compared to the individuals having only a late CMI response to Ty21a. Thus, vaccine immunogenicity, and perhaps even efficacy, could potentially be related to resident microbiota community structure. While the sample size is relatively small, and a larger cohort study is clearly warranted, these initial observations have important implications for the role the gut microbiota play in vaccine efficacy and will guide future studies to evaluate the interplay between the immune system and gut microbiota following immunization and/or challenge with wild-type *S.* Typhi. These results provide a unique view into the dramatic heterogeneity of the fecal microbiota and the host immune system among individuals.

## Materials and Methods

### Volunteer recruitment and sample collection

Stool samples and peripheral blood mononuclear cells (PBMC) were collected from healthy adult volunteers recruited from the Baltimore-Washington area and University of Maryland, Baltimore campus (Table S1). Written informed consent was obtained and all procedures were approved by the University of Maryland, Baltimore IRB (IRB #s HP-00040022 and HP-00040025). Samples were collected a week prior to immunization (day -7), the day of immunization (day 0), and at 8 time-points after immunization (days 2, 4, 7, 10, 14, 28, 42, 56). Approximately 0.5–2 grams of stool were immediately mixed with RNALater and stored at −80°C until further processing. PBMC were isolated immediately after blood draws by density gradient centrifugation and cryopreserved in liquid nitrogen following standard techniques [31].

### DNA extraction and pyrosequencing of barcoded 16S rRNA gene amplicons

Total DNA was extracted from 0.2 grams using the ZR Fecal DNA isolation kit (cat. #D6010, ZYMO Research Corp.) with modifications including an enzymatic pre-treatment with mutanolysin (5000 units ml^−1^, Sigma cat. #M9901) and lysozyme (100 mg ml^−1^, Sigma cat. #L-6876) in conjunction with aggressive bead beating using Lysing Matrix tubes (QBiogene) and a FastPrep FP120 instrument (Qbiogene). Barcoded primers [32] were used to amplify the bacterial 16S rRNA gene region 27F-338R from 50 ng of purified DNA using AccuPrime High Fidelity DNA polymerase (Invitrogen) in a total reaction volume of 25 μL. Reactions were run in a PTC-100 thermal controller (MJ Research) using the following cycling parameters: 5 min of denaturation at 95°C, followed by 25 cycles of 30 s at 95°C, 30 s at 55°C, and 60 s at 68°C, with a final extension at 72°C for 7 min. Negative controls without a DNA template were included for each barcoded primer pair. Amplicons were quantified using the Quant-iT PicoGreen dsDNA assay and equimolar amounts (100 ng) of the PCR product were mixed in a single tube. Amplification primers and reaction buffer were removed from each sample using the AMPure Kit (Agencourt). The purified amplicon mixtures were sequenced by 454 pyrosequencing using 454 Life Sciences primer A by the Genomics Resource Center at the Institute for Genome Sciences, University of Maryland School of Medicine.

### 16S rRNA sequence processing and analysis

Sequences generated from pyrosequencing of bacterial 16S rRNA gene amplicons were processed using mothur [18], [33]. Briefly, sequences were denoised, trimmed, quality and chimera-checked using *de novo* uchime, and clustered into operational taxonomic units (OTUs) at 97% pairwise identity, with representative sequences aligned to the Silva reference alignment [34]. Taxonomic classifications were assigned using the naïve Bayesian classifier with the Greengenes database (downloaded March 2011) [35]. Rarefied OTUs (randomly sub-sampled to normalize sequence counts) were used to calculate community richness, evenness, and diversity for each sample over time. The partitioning around medoids (pam) clustering algorithm was used as described by Arumugam and colleagues [19] with the Jensen-Shannon divergence of the classified genus-level abundance profiles. Multivariate kernel density estimation and principle coordinate analysis (PCoA) with visualizations were performed using the R packages feature and vegan, respectively [36], [37]. Dot plots of Jensen-Shannon divergence values were generated using a custom Perl script [38].

### *In vitro* stimulation with *S.* Typhi antigens and intracellular cytokine/chemokine detection

As previously described [10], PBMC were incubated with *S*. Typhi-infected stimulators followed by staining for 13-color flow cytometry analysis to identify IL-17A, IL-2, IFN-γ, TNF-α, and MIP-1β. MIP-1β staining was not performed for volunteers 48s and 50s due to unavailability of the antibody. Flow cytometric data were obtained using an LSRII flow cytometer (BD, Franklin Lakes, NJ) and analyzed using the WinList version 7 software package (Verity Software House, Topsham, ME). Results are presented as net values, with the background from uninfected stimulators subtracted, since low levels of cytokine production were detectable following stimulation with uninfected stimulators. Net increases of >1% cytokine positive cells over baseline responses at day 0 were found to be statistically significant (chi-square test; p<0.01).

### Antibody measurements

Levels of serum IgA and IgG against *S*. Typhi LPS were measured by ELISA using previously described methods [39]. Post-vaccination fold increases of anti-LPS antibody titers were calculated as titers post-vaccination divided by the corresponding pre-vaccination titers multiplied by 100. Sero-conversion was defined as a ≥4-fold increase in post-vaccination antibody titer at any time point post-vaccination compared to pre-vaccination (Table S2).

### Statistical methodologies

Categorical analysis to identify significantly different bacterial groups between the three multiphasic CMI responders and late CMI responders was performed using metastats [25]. Other statistical methods were implemented using the JMP statistical software package (Version 9, SAS Institute, Cary, NC).

### Nucleotide accession numbers

The 16S rRNA 454 pyrosequencing data has been deposited in the GenBank Sequence Read Archive under accession number SRA053563.

## Supporting Information

Figure S1 **Ternary plot for the relative abundances of Prevotella, Bacteroides, and Firmicutes among all samples.** Samples are colored by the Bacteroides-dominated ‘community type’ (n = 123) in blue and the Prevotella-dominated ‘community type’ (n = 39) in red as determined by the partitioning around medoids (pam) clustering algorithm.
(TIF)

Figure S2 **PCoA plots as in** **Figure 2A** **with black lines connecting sequential samples.** The Jensen-Shannon divergence for (A) all samples and (B) four representative individuals colored by the multivariate kernel density estimation with vaccination groups designated by unvaccinated control (▪), one dose Ty21a (•), and four dose Ty21a (Δ).
(TIF)

Figure S3 **Community diversity between humoral ‘responders’ and ‘non-responders.’** Sero-conversion was defined as a ≥4-fold increase in post-vaccination antibody titer at any time point post-vaccination compared to pre-vaccination. Shannon diversity, inverted Simpson diversity, and phylogenetic diversity (PD) were calculated from the rarefied OTU data. The bars represent the mean diversity values. In all measures, the ‘responders’ were not found to significantly differ compared to the ‘non-responders.’
(TIF)

Table S1 **Clinical study design and volunteer information.** Stool and PBMC samples were collected from a total of four unvaccinated volunteers, seven volunteers receiving one-dose, and six volunteers receiving four-doses of the oral vaccine Ty21a.
(DOCX)

Table S2 **Serum antibody responses.** Titers of serum IgA and IgG against *S*. Typhi LPS for (**A**) unvaccinated volunteers, (**B**) volunteers receiving one-dose Ty21a, and (**C**) volunteers receiving four-doses Ty21a. Values ≥4-fold increase are highlighted in gray. DPI, days post-immunization.
(DOCX)

Table S3 **CMI responses (IFN-γ production) in Ty21a vaccinees and controls.** Significant increases in CD8+ IFN-γ responses are highlighted in gray. DPI, days post-immunization.
(DOCX)

Table S4 **Differentially abundant OTUs in multiphasic CMI responders and late responders.** A q-value cutoff of q<0.05 was used.
(DOCX)
