# Supplementary figures and images for: Impact of Oral Typhoid Vaccination on the Human Gut Microbiota and Correlations with S. Typhi-Specific Immunological Responses
Source: PLoS One. 2013 Apr 24;8(4):e62026. doi: 10.1371/journal.pone.0062026 (PMC3634757; doi:10.1371/journal.pone.0062026)

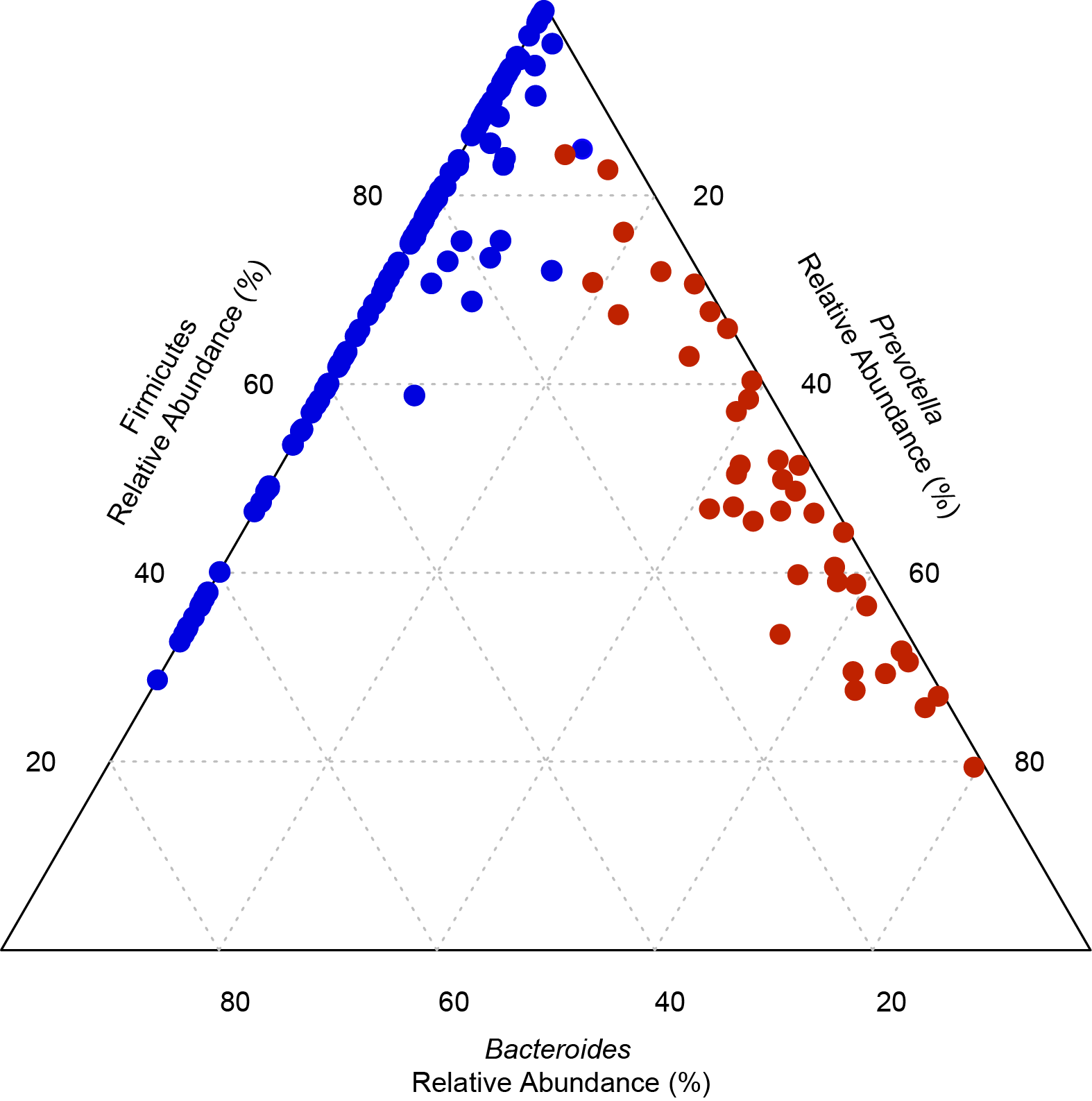

Supplement: Figure S1 — Ternary plot for the relative abundances of Prevotella, Bacteroides, and Firmicutes among all samples. Samples are colored by the Bacteroides-dominated ‘community type’ (n = 123) in blue and the Prevotella-dominated ‘community type’ (n = 39) in red as determined by the partitioning around medoids (pam) clustering algorithm. (TIF) [file pone.0062026.s001.tif]

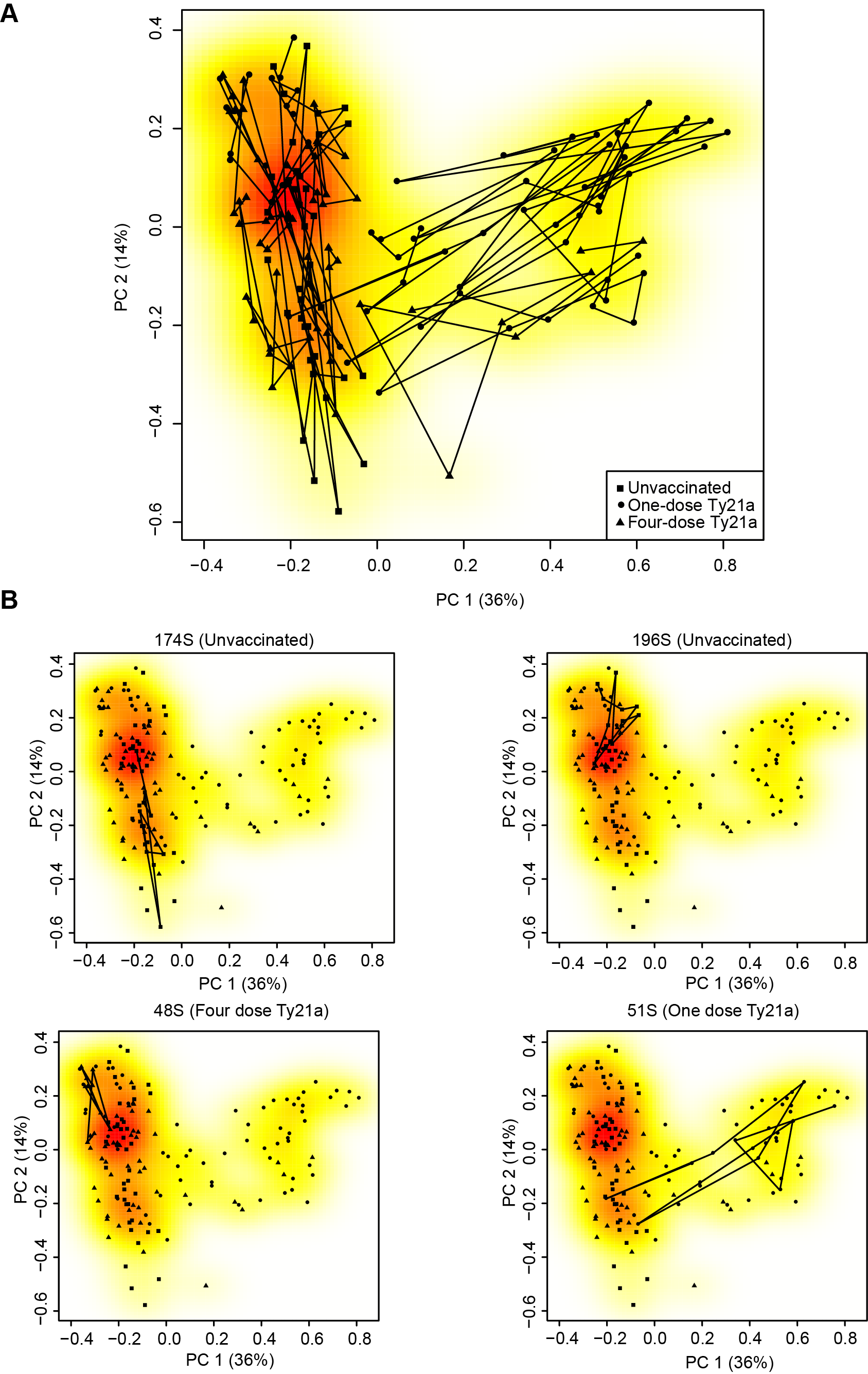

Supplement: Figure S2 — PCoA plots as in Figure 2A with black lines connecting sequential samples. The Jensen-Shannon divergence for (A) all samples and (B) four representative individuals colored by the multivariate kernel density estimation with vaccination groups designated by unvaccinated control (▪), one dose Ty21a (•), and four dose Ty21a (Δ). (TIF) [file pone.0062026.s002.tif]

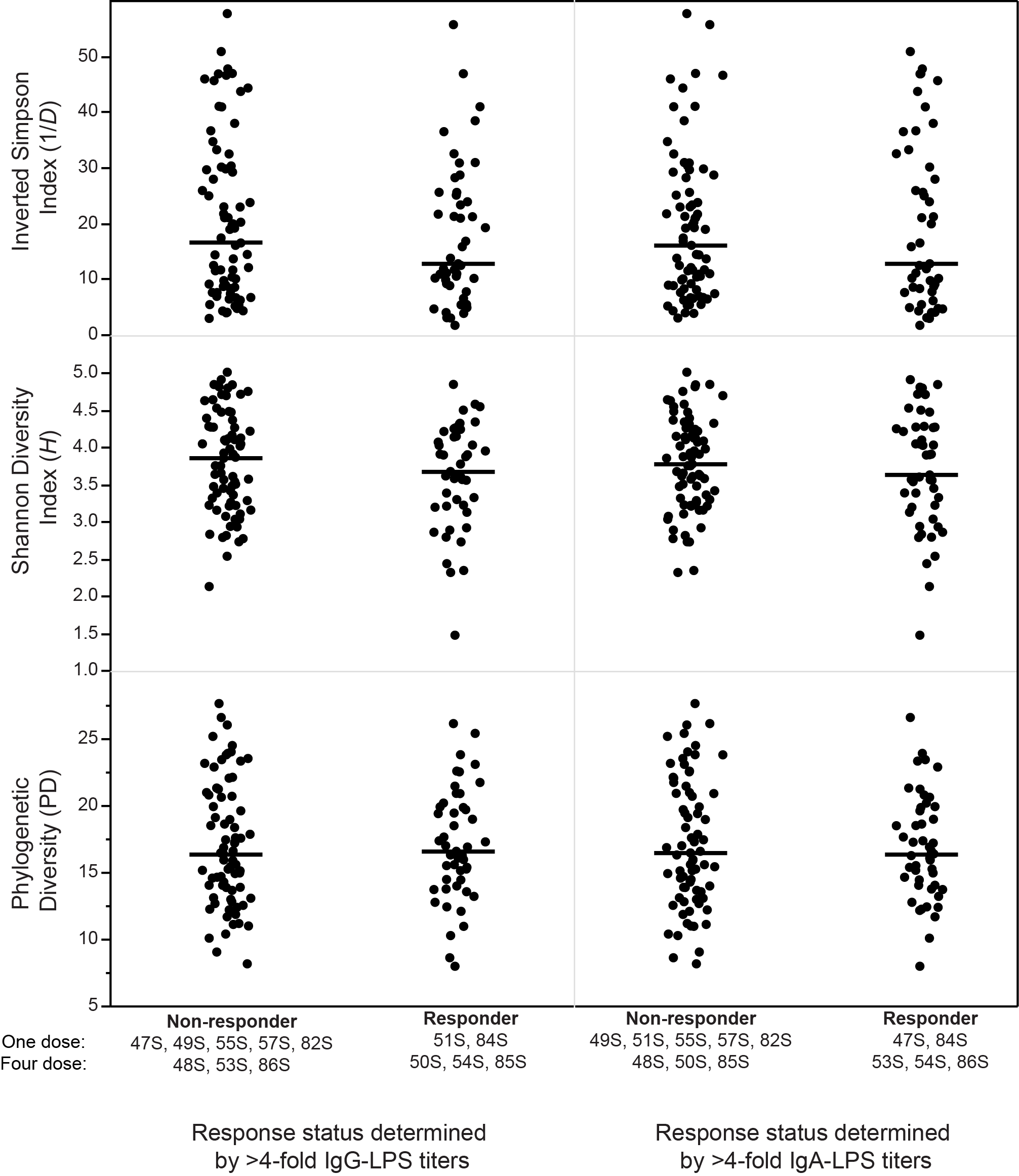

Supplement: Figure S3 — Community diversity between humoral ‘responders’ and ‘non-responders.’ Sero-conversion was defined as a ≥4-fold increase in post-vaccination antibody titer at any time point post-vaccination compared to pre-vaccination. Shannon diversity, inverted Simpson diversity, and phylogenetic diversity (PD) were calculated from the rarefied OTU data. The bars represent the mean diversity values. In all measures, the ‘responders’ were not found to significantly differ compared to the ‘non-responders.’ (TIF) [file pone.0062026.s003.tif]
