# Supplementary material for: Impact of Oral Typhoid Vaccination on the Human Gut Microbiota and Correlations with S. Typhi-Specific Immunological Responses
Source: PLoS One. 2013 Apr 24;8(4):e62026. doi: 10.1371/journal.pone.0062026 (PMC3634757; doi:10.1371/journal.pone.0062026)
Supplement: Table S1 — Clinical study design and volunteer information. Stool and PBMC samples were collected from a total of four unvaccinated volunteers, seven volunteers receiving one-dose, and six volunteers receiving four-doses of the oral vaccine Ty21a. (DOCX) [file pone.0062026.s004.docx]

**Table S1.** **Clinical study design and volunteer information.** Stool and PBMC samples were collected from a total of four unvaccinated volunteers, seven volunteers receiving one-dose, and six volunteers receiving four-doses of the oral vaccine Ty21a.

| **Volunteer** | **Vaccine schedule** | **Gender** | **Age** | **Stool collected (days)** | **PBMC collected (days)** |
| --- | --- | --- | --- | --- | --- |
| 174S | Unvaccinated | F | 45 | -7, 0, 2, 4, 7, 10, 14, 28, 42 | 0, 2, 4, 7, 10, 14, 28, 42, 56 |
| 177S | Unvaccinated | F | 23 | -7, 0, 2, 4, 7, 10, 14, 28, 42, 56 | 0, 2, 4, 7, 10, 14, 28, 42, 56 |
| 196S | Unvaccinated | M | 26 | -7, 0, 2, 4, 7, 10, 14, 28, 42, 56 | 0, 2, 7, 10, 14, 28, 42, 56 |
| 198S | Unvaccinated | M | 26 | -7, 0, 2, 4, 7, 10, 14, 28, 42, 56 | 0, 2, 4, 7, 10, 14, 28, 42, 56 |
| 47S | One-dose Ty21a | M | 27 | -7, 0, 2, 4, 7, 10, 14, 28, 42, 56 | 0, 2, 4, 7, 10, 14, 28, 42, 56 |
| 49S | One-dose Ty21a | M | 36 | -7, 0, 4, 7, 10, 14, 28, 42, 56 | 0, 2, 4, 7, 10, 14, 28, 42, 56 |
| 51S | One-dose Ty21a | M | 50 | -7, 0, 2, 4, 7, 10, 14, 28, 42, 56 | 0, 2, 4, 7, 10, 14, 28, 42, 56 |
| 55S | One-dose Ty21a | M | 40 | -7, 0, 2, 4, 7, 10, 14, 28, 42, 56 | 0, 2, 4, 7, 10, 14, 28, 42, 56 |
| 57S | One-dose Ty21a | M | 39 | -7, 0, 2, 4, 7, 10, 14, 28, 42, 56 | 0, 2, 4, 7, 10, 14, 28, 42, 56 |
| 82S | One-dose Ty21a | F | 26 | -7, 0, 2, 4, 7, 10, 14, 28, 42 | 0, 2, 4, 7, 10, 14, 28, 42, 56 |
| 84S | One-dose Ty21a | F | 21 | -7, 0, 2, 4, 7, 10, 14, 28, 42, 56 | 0, 2, 4, 7, 10, 14, 28, 42, 56 |
| 48S | Four-dose Ty21a | M | 25 | -7, 0, 2, 4, 7, 10, 14, 28, 42, 56 | 0, 2, 4, 7, 10, 14, 28, 42, 56 |
| 50S | Four-dose Ty21a | F | 25 | -7, 0, 2, 4, 7, 10, 14, 28, 42, 56 | 0, 2, 4, 7, 10, 14, 28, 42, 56 |
| 53S | Four-dose Ty21a | F | 26 | -7, 0, 2, 4, 7, 10, 14, 28, 42, 56 | 0, 2, 4, 7, 10, 14, 28, 42, 56 |
| 54S | Four-dose Ty21a | M | 31 | -7, 0, 2, 4, 7, 14, 28, 42 | 0, 2, 4, 7, 10, 14, 28, 42, 56 |
| 85S | Four-dose Ty21a | F | 19 | -7, 0, 2, 4, 7, 10, 14, 28, 56 | 0, 2, 4, 7, 10, 14, 28, 42, 56 |
| 86S | Four-dose Ty21a | F | 19 | -7, 0, 2, 4, 7, 10, 14, 42 | 0, 2, 4, 7, 10, 14, 28, 42, 56 |
