# Supplementary material for: Impact of Oral Typhoid Vaccination on the Human Gut Microbiota and Correlations with S. Typhi-Specific Immunological Responses
Source: PLoS One. 2013 Apr 24;8(4):e62026. doi: 10.1371/journal.pone.0062026 (PMC3634757; doi:10.1371/journal.pone.0062026)
Supplement: Table S2 — Serum antibody responses. Titers of serum IgA and IgG against S. Typhi LPS for (A) unvaccinated volunteers, (B) volunteers receiving one-dose Ty21a, and (C) volunteers receiving four-doses Ty21a. Values ≥4-fold increase are highlighted in gray. DPI, days post-immunization. (DOCX) [file pone.0062026.s005.docx]

**Table S2. Serum antibody responses.** Titers of serum IgA and IgG against *S*. Typhi LPS for (**A**) unvaccinated volunteers, (**B**) volunteers receiving one-dose Ty21a, and (**C**) volunteers receiving four-doses Ty21a. Values ≥ 4-fold increase are highlighted in gray. DPI, days post-immunization.

**A**

| **Control** | **174S** | | **177S** | | **196S** | | **198S** | |
| --- | --- | --- | --- | --- | --- | --- | --- | --- |
| **DPI** | **IgA LPS** | **IgG LPS** | **IgA LPS** | **IgG LPS** | **IgA LPS** | **IgG LPS** | **IgA LPS** | **IgG LPS** |
| **2** | 1.3 | 0.9 | 0.8 | 1.2 | 1.3 | 1.1 | 1.1 | 0.9 |
| **4** | 1.3 | 0.8 | 1.2 | 2.0 | 1.3 | 0.9 | 1.0 | 1.1 |
| **7** | 1.2 | 0.9 | 1.0 | 1.6 | 1.2 | 0.9 | 0.9 | 0.8 |
| **10** | 1.4 | 1.3 | 1.0 | 0.9 | 0.9 | 0.9 | 0.5 | 0.6 |
| **14** | 1.4 | 1.0 | 0.9 | 1.0 | 1.0 | 0.8 | 0.7 | 0.9 |
| **28** | 1.8 | 1.1 | 0.8 | 1.5 | 1.1 | 1.0 | 0.8 | 0.8 |
| **42** | 1.6 | 1.1 | 0.8 | 1.2 | 1.1 | 1.0 | 0.7 | 0.8 |
| **56** | 1.2 | 1.3 | 0.9 | 1.2 | 0.9 | 1.0 | 0.8 | 0.7 |

**B**

| **1-dose** | **47S** | | **49S** | | **51S** | | **55S** | |
| --- | --- | --- | --- | --- | --- | --- | --- | --- |
| **DPI** | **IgA LPS** | **IgG LPS** | **IgA LPS** | **IgG LPS** | **IgA LPS** | **IgG LPS** | **IgA LPS** | **IgG LPS** |
| **2** | 1.0 | 1.1 | 1.0 | 0.9 | 1.0 | 1.1 | 0.9 | 0.9 |
| **4** | 0.9 | 0.8 | 0.9 | 1.0 | 1.1 | 1.1 | 0.9 | 0.9 |
| **7** | **96.2** | 2.8 | 1.0 | 2.1 | 2.1 | 3.4 | 1.9 | 1.0 |
| **10** | **28.6** | 3.9 | 1.1 | 2.7 | 2.8 | **8.0** | 1.1 | 1.4 |
| **14** | **16.2** | 3.1 | 1.1 | 2.6 | 2.1 | **5.4** | 1.1 | 1.3 |
| **28** | 1.0 | 3.2 | 0.8 | 2.5 | 1.5 | **5.6** | 1.1 | 1.6 |
| **42** | 1.0 | 3.2 | 0.7 | 1.4 | 1.7 | **4.3** | 1.0 | 1.3 |
| **56** | 0.9 | 2.0 | 0.7 | 1.4 | 1.6 | 2.8 | 0.8 | 1.0 |

| **1-dose** | **57S** | | **82S** | | **84S** | |
| --- | --- | --- | --- | --- | --- | --- |
| **DPI** | **IgA LPS** | **IgG LPS** | **IgA LPS** | **IgG LPS** | **IgA LPS** | **IgG LPS** |
| **2** | 0.9 | 0.8 | 0.8 | 1.0 | 1.1 | 0.9 |
| **4** | 0.7 | 0.7 | 0.6 | 0.5 | 0.9 | 0.9 |
| **7** | 1.6 | 1.1 | 0.6 | 0.7 | **13.3** | **13.0** |
| **10** | 2.7 | 1.5 | 0.5 | 0.8 | **17.0** | **33.8** |
| **14** | 3.1 | 1.6 | 0.5 | 1.1 | **9.5** | **15.0** |
| **28** | 1.4 | 1.5 | 0.2 | 1.0 | 2.2 | **7.1** |
| **42** | 1.0 | 1.1 | 0.2 | 0.8 | 1.5 | 3.9 |
| **56** | 1.0 | 0.9 | 0.2 | 0.7 | 1.1 | 2.7 |

**C**

| **4-dose** | **48S** | | **50S** | | **53S** | | **54S** | | **85S** | | **86S** | |
| --- | --- | --- | --- | --- | --- | --- | --- | --- | --- | --- | --- | --- |
| **DPI** | **IgA LPS** | **IgG LPS** | **IgA LPS** | **IgG LPS** | **IgA LPS** | **IgG LPS** | **IgA LPS** | **IgG LPS** | **IgA LPS** | **IgG LPS** | **IgA LPS** | **IgG LPS** |
| **2** | 0.9 | 0.9 | 0.9 | 1.0 | 0.9 | 1.0 | 1.0 | 1.0 | 1.0 | 0.8 | 1.0 | 0.6 |
| **4** | 0.8 | 1.1 | 0.2 | 1.1 | 0.8 | 0.9 | 0.9 | 1.2 | 0.8 | 0.9 | 1.2 | 0.6 |
| **7** | 1.7 | 1.0 | 0.2 | **8.4** | **13.5** | 0.9 | **10.2** | **8.6** | 2.1 | **4.2** | 3.1 | 1.9 |
| **10** | 3.3 | 1.1 | 0.4 | **42.4** | **16.4** | 1.0 | **14.5** | **103.3** | 2.6 | **5.7** | 3.9 | 2.9 |
| **14** | 1.8 | 1.1 | 0.4 | **50.7** | **7.6** | 1.4 | **11.1** | **53.7** | 2.0 | 3.8 | **20.7** | 3.7 |
| **28** | 0.7 | 1.1 | 0.4 | **18.9** | 1.8 | 1.2 | 2.4 | **25.3** | 1.6 | 2.8 | 3.7 | 2.4 |
| **42** | 1.0 | 1.2 | 0.4 | **13.2** | 1.3 | 1.1 | 1.9 | **11.8** | 1.3 | 1.9 | 2.5 | 1.5 |
| **56** | 1.1 | 1.0 | 0.4 | **16.9** | 1.1 | 1.0 | 1.9 | **9.4** | 1.2 | 1.5 | 1.2 | 1.3 |
