# Supplementary material for: Impact of Oral Typhoid Vaccination on the Human Gut Microbiota and Correlations with S. Typhi-Specific Immunological Responses
Source: PLoS One. 2013 Apr 24;8(4):e62026. doi: 10.1371/journal.pone.0062026 (PMC3634757; doi:10.1371/journal.pone.0062026)
Supplement: Table S3 — CMI responses (IFN-γ production) in Ty21a vaccinees and controls. Significant increases in CD8+ IFN-γ responses are highlighted in gray. DPI, days post-immunization. (DOCX) [file pone.0062026.s006.docx]

**Table S3. CMI responses (IFN-γ** **production) in Ty21a vaccinees and controls.** Significant increases in CD8+ IFN-γ responses are highlighted in gray. DPI, days post-immunization.

|  | 4-Dose | | | | | | Control | |
| --- | --- | --- | --- | --- | --- | --- | --- | --- |
| **DPI** | **48S** | **50S** | **53S** | **54S** | **85S** | **86S** | **177S** | **196S** |
| **2** | 0 | 0 | 0 | 0 | 0 | **1.6** | 0 | 0.59 |
| **4** | 0.67 | **3.86** | **2.77** | 0.17 | 0.48 | 0.57 | 0.52 | 0 |
| **7** | 0 | **2.1** | **1.74** | 0 | 0.11 | 0.69 | 0.03 | 0 |
| **10** | 0 | **2.17** | **2.24** | 0 | 0 | **1.36** | 0.61 | 0 |
| **14** | 0.49 | **3.22** | **1.16** | **2.28** | 0 | 0.87 | 0.33 | 0 |
| **28** | 0.38 | **2.49** | 0.99 | **3.8** | 0 | **1.16** | 0.04 | 0 |
| **42** | 0 | **1.63** | **4.36** | **4.64** | **2.32** | 0.67 | 0.27 | 0 |
| **56** | 0.07 | **2.27** | **3.87** | **3.44** | 0.05 | **2.2** | 0.36 | 0 |
